# Proceedings of the Galesburg Medical Society

**Published:** 1860-10

**Authors:** M. K. Taylor, H. M. Starkloff

**Affiliations:** President; Secretary


### PROCEEDINGS

### OF THE MEDICAL SOCIETY OF THE CITY OF GALESBURG, HELD SEPTEMBER, 8TH, 1860.

At a regular meeting of the Galesburg City Medical Society held September, 8th, 1860, the following report of a committee appointed at a previous session, was received and adopted unanimously, and copies ordered to be furnished to the *Chicago Medical Journal* and the *Chicago Medical Examiner*, for publication:

Your committee to whom was referred the several subjects relating to the necessities for some legislation favoring the study of Practical Anatomy, by students and members of the medical profession, for a registry law of births, marriages, and deaths, and for the extension to medical men of the privilege of witholding, when called as witnesses, any information derived in the exercise of their professional duties, and which was necessary to the proper discharge thereof, submit the following preamble and resolutions, as expressive of the sense of this Society.

WHEREAS, In the study of the science of medicine and surgery, and to a proper understanding and practice of the same it is indispensably necessary that the student have an opportunity to become familiar with the science of *Human Anatomy*, by practical dissections with his own hands, and

WHEREAS, Such course of study is held as a pre-requisite to graduation in all well regulated medical schools, and is also held by the public to be of paramount importance to a thorough medical education; and

WHEREAS, Both the public and the law hold medical men pecuniarily and morally responsible for a reasonable knowledge of this science in the exercise of their vocation; and

WHEREAS, The study of Human Anatomy cannot now be pursued in the State, except in direct violation of the laws thereof, and the liability to severe fines and penalties, and

WHEREAS, We believe this inconsistency and injustice of the law in the requirement of the exercise of this knowledge under penalty, which, at the same time, under like penalty, it prohibits being obtained, should be removed; and

WHEREAS, We believe that a proper registration of births, marriages, and deaths, is necessary in a legal, medical, and sanitary point of view, and will conduce greatly to the general welfare of the inhabitants and the advancement of the industrial interests of the State. And

WHEREAS, By the common law of the land, and by the rulings of the courts in this State, physicians and surgeons may be made to disclose any information acquired confidentially in their attendance professionally upon any person, no matter how repugnant to their sense of propriety, honor, and morality, such disclosures may be; therefore,

*Resolved*, That we deem it wise and necessary, on the part of the Legislature of this State, to make some provision which will admit students of medicine and members of the profession to supply themselves with materiel from subjects interred at the public expense, when not claimed by relations or friends, under such restrictions for the protection of the public interests and feelings as the delicate nature of the case may suggest.

*Resolved*, That a law for the registration of births, marriages, and deaths, will contribute much to a better understanding of our climate, vital statistics, sanitary and industrial condition; to say nothing of the legal, historical, and medical knowledge otherwise accruing therefrom.

*Resolved*, That we hold the relation of physician and patient to be of the most private, personal, and confidential character; and as such should ever be held inviolate, and as exempt from inquisitorial proceedings as those of the attorney and his client. That in accordance with these sentiments we claim that no person duly authorised to practice medicine and surgery should be compelled to disclose any information which he may have acquired in attendance on any patient in a professional character, and which information was necessary to enable him to prescribe intelligently, as a physician, or do any act as a surgeon.

*Resolved*, That the statutes of the States of New York, Michigan, Iowa, Wisconsin, and Missouri, extending to the medical profession these legal privileges, are founded in justice and good morals, and have a proper regard for the peace of families and the community, and therefore receive our entire approval in meeting the wants of the profession in this State.

*Resolved*, That at the proper time this Society will prepare a memorial to the Legislature calling attention to these, as we believe, rightful statutory provisions, and ask for such enactments as the several cases herewith presented may require.

*Resolved*, That we recommend similar action to other medical societies, and we solicit their active co-operation in carrying out the spirit of these resolutions.

M. K. TAYLOR, M. D., President.

H. M. STARKLOFF, M. D., Secretary.

☞ We take pleasure in returning our thanks to those of our patrons who have promptly responded to the bills forwarded with the last number of the *Journal*, and whose names appear in the present issue. We trust we shall not be forgotten by those who have not yet paid, and that the October list of receipts may be more ample than that for the preceeding month.

☞ The Illinois State Medical Society holds its Annual Meeting for the year 1861, on the first Tuesday of May next, in the city of Jacksonville.

☞ Prof. J. Adams Allen, of Rush Medical College, has been appointed to the Chair of Materia Medica, in the Chicago College of Pharmacy. The latter appointment does not interfere with his teaching in the Medical College.

